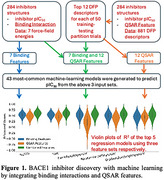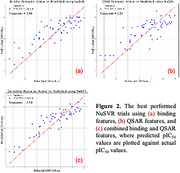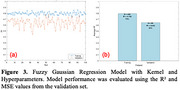# Enhancing BACE1 Inhibitor Discovery with Machine Learning by Integrating Binding Interactions and QSAR Features

**DOI:** 10.1002/alz70859_100446

**Published:** 2025-12-25

**Authors:** Tianhua Zhai, David Shen, Magally Arevalo Navarro, Zuyi Jacky Huang, Li Shen

**Affiliations:** ^1^ Institute for Biomedical Informatics, University of Pennsylvania, Philadelphia, PA USA; ^2^ Harriton High School, Bryn Mawr, PA USA; ^3^ Villanova University, Villanova, PA USA

## Abstract

**Background:**

Beta‐secretase 1 (BACE1) plays a crucial role in the formation of neurotoxic amyloid‐beta peptides in Alzheimer's disease. Effective BACE1 inhibitors are urgently needed for AD intervention. This study utilized available BACE1 inhibitor data to develop a machine‐learning approach that integrates both BACE1‐ligand interaction features and inhibitor QSAR descriptors to discover new BACE1 inhibitors.

**Method:**

Ligand‐protein interactions of 284 BACE1‐ligand complexes, along with experimental pIC50 values (indicating binding affinities), were obtained from the Protein Data Bank. Docking simulations were performed using Molsoft ICM software to reproduce the binding conformation of each BACE1‐ligand complex, yielding binding interaction features (seven force‐field energy components, such as hydrogen bonding and hydrophobic interactions). 881 QSAR features for each ligand were extracted using the PubChem molecular fingerprint calculator. Subsequently, 12 highly correlated QSAR features were selected via statistical tests on training data. Finally, 43 machine‐learning models were developed to predict pIC50 using three input sets: 1) binding interaction features, 2) the top 12 QSAR features identified, and 3) the combined 19 binding and QSAR features. Each model was trained on 80% of the data and tested on 20%, with performance evaluated on 100 random partitions using the R² metric.

**Result:**

The top 5 machine‐learning models demonstrated improved prediction accuracy when combining binding and QSAR features compared to using either feature set alone (Figure 1). The NuSVR model achieved the best prediction performance. In the best‐performing trials, NuSVR achieved R² = 0.78 using combined features, compared to R² = 0.65 and R² = 0.64 using binding and QSAR features, respectively (Figure 2). Additionally, the fuzzy Gaussian regression model with seven binding interaction features and molecular weight also demonstrated acceptable performance, with R² = 0.642 and mean squared error (MSE) = 0.45 (Figure 3).

**Conclusion:**

Machine‐learning models integrating binding and QSAR features enhance the prediction of BACE1‐ligand binding affinity (pIC50), enabling efficient virtual screening for potent BACE1 inhibitors. These advancements offer potential for accelerating drug discovery for Alzheimer’s disease. Future work will expand these models to include broader chemical databases, furthering intervention strategies for AD.